# Commuting Mode Choice in a High-Density City: Do Land-Use Density and Diversity Matter in Hong Kong?

**DOI:** 10.3390/ijerph15050920

**Published:** 2018-05-04

**Authors:** Yi Lu, Guibo Sun, Chinmoy Sarkar, Zhonghua Gou, Yang Xiao

**Affiliations:** 1 Department of Architecture and Civil Engineering, City University of Hong Kong, Kowloon Tong, Hong Kong, China; 2 City University of Hong Kong Shenzhen Research Institute, Shenzhen 518057, China; 3 Faculty of Architecture, The University of Hong Kong, Pokfulam, Hong Kong, China; gbsun@hku.hk (G.S.); csarkar@hku.hk (C.S.); 4 School of Engineering and Built Environment, Griffith University, Gold Coast, QLD 4215, Australia; z.gou@griffith.edu.au; 5 Department of Urban Planning, Tongji University, Shanghai 200092, China; yxiao@tongji.edu.cn

**Keywords:** travel choice, commuting trips, built environment, high density, land use policy, urban design

## Abstract

Hong Kong is a densely populated and transit-oriented Chinese city, which provides an ideal urban environment with which to study the various successful facets of land use policy as a model for potential replication to curb increasing car use in other Chinese cities. We examine the commuting mode choice of 203,900 households living in 4768 street blocks in Hong Kong from 2011 census. A street block is the smallest planning unit, made up of one or more housing estates with a homogenous built environment and socioeconomic status. The built environment is measured using the five *Ds* framework, an international dimensioning framework for classifying and measuring attributes of the built environment for physical activity and travel behaviors. Generalized, multi-level mixed models were applied to detect the associations between travel choice and built environment characteristics, while adjusting for socioeconomic status. Design and destination accessibility had greater effects on the choices to walk and take public transport than on the choice to drive. Density and diversity had only marginal effects on mode choice. Unexpectedly, distance to the urban center had the opposite effect on automobile use to that found in Western studies. Hong Kong residents living close to the urban center were more likely to drive for commuting trips. The contrasting findings between our study and Western studies suggest that the associations between a high-density built environment and travel choice vary with urban context.

## 1. Introduction 

Numerous studies have suggested that certain built environment features such as a better job−housing balance and greater street connectivity can increase the propensity to walk and use public transport for commuting trips and therefore stimulate physical activity, improve air quality, and reduce traffic congestion [1,2,3,4,5,6,7,8,9,10,11]. 

China is experiencing rapid urbanization and motorization [12]. The urban population increased to 792 million in 2016 from 191 million in 1980, primarily as a result of rural-to-urban migration [13]. However, the outdated urban planning has largely failed to keep pace with this ongoing significant demographic shift, resulting in unsustainable land use with insufficient pedestrian infrastructure and pedestrian-oriented destinations, and immense block sizes (and hence longer trip lengths) in many new urban developments. Furthermore, the expansion of the prosperous middle class has resulted in soaring auto ownership and mode shifts toward inactive commuting and sedentary lifestyles, which may partially explain the rapid drop in physical activity and an increased risk of chronic diseases among urban residents [14,15]. The integration of land use policy and public transportation systems, such as in transit-oriented development, has been implemented or is under consideration in many high-density Chinese cities. Planning practice and policymaking in this area, however, depend heavily on empirical evidence from Western countries. To bridge this gap, this study tries to reveal the relationship between mode choice and the built environment in a high-density Chinese city. 

The 5*Ds* framework is an international dimensioning framework that provides order-of-magnitude insights into the built environment [16,17]. It is an effective framework for measuring and classifying attributes of the built environment to test scientific hypotheses in travel behavior studies. The 5Ds are density, assessed as variables of interest in a unit of area; diversity, which pertains to the proportion of different land use types; design, measured as street patterns and related design features; destination accessibility, measured as the proximity to common trip destinations; and distance to transit, measured as the proximity to transit stops.

1. *Density*. In neighborhoods with high residential density, destinations are often closer to a person’s home and therefore they can be reached by walking (Sun et al., 2016). Researchers have also argued that density is likely to be a proxy for low-income populations, better public transportation service, or low levels of car ownership [17,18].
2. *Diversity*. Having diversified land use classes in the neighborhood can also promote walking and taking public transportation [19]. Diversity is often measured by the entropy value [20]. The job−housing balance—the ratio of employment to the residential population—is also alternatively used as a simpler measure (Rajamani et al., 2003; Bento et al., 2005).
3. *Design*. Design includes the connectivity and quality of sidewalks [21]. More sophisticated measures of street-level configuration such as movement potential or network analysis have also been used in active travel and health studies [22,23]. Some studies have demonstrated the link between continuous sidewalks and a grid-like street pattern and propensity to walk or take public transit [3,4,17,24]. However, studies conducted in Beijing have found that a higher density of main roads increases the possibility of commuting by car [25].
4. *Destination accessibility*. The distance to city center is often used to assess destination accessibility. The workplaces in many cities in China are largely located in traditional urban centers than at peripheries despite three decades of urban sprawl [26]. Residents are less prone to drive to their workplaces if living close to a city center [20,27].
5. *Distance to transit.* Proximity to the nearby transit stop is positively associated with residents’ propensity to make transit trips [28,29,30].

One recent meta-analyses of the travel behavior-built environment association in Western cities has used elasticity values to evaluate the impact’s magnitude of a given factor on a desired outcome [20]. Elasticity is measured as the rate of change in an outcome as a result of 1% change in a factor. For example, if a 1% increase in population density results in increased probability of walking by 0.39%, then the elasticity of the effect of population density on walking is 0.39. Ewing and Cervero revealed that car use was highly associated with destinations accessibility (elasticity estimates with respect to job accessibility −0.20, distance to the urban center −0.22) and street network design (intersection density −0.12). In their study, walking was strongly associated with land use diversity (land-use mix 0.15) and intersection density (0.39). Transit use was strongly related to distance to nearest transit stop (0.29) and intersection density (0.23), and secondarily related to land use diversity (0.12). The elasticity values also make it possible to compare the findings in the current studies with those in Western cities.

Our study focuses on Hong Kong, an iconic high-density city in China. Hong Kong and other high-density cities in China are different from cities in Western countries in many respects, having larger populations, higher population densities, lower crime rates, a lower socioeconomic status, and different family structures and other cultural affinities [31,32]. For example, the dwelling density of the Hong Kong urban area is over 1250 units per hectare, which is 5 times higher than that of a typical relatively dense European city (Cerin et al., 2011). Some recent empirical studies conducted in China showed mixed results about the association of the 5Ds framework and travel behaviors [33,34,35,36,37]. Hence, replicating policies based on Western contexts in Chinese cities may prove to be ineffective or misleading. The unique characteristics of land use development patterns, especially urban density and land use diversity, a highly evolved public transport system, and a strong similarity in population characteristics to mainland China makes Hong Kong a classic test bed for investigating hypotheses on the links between land use and travel behavior. It provides an ideal urban context with which to study the various successful facets of land use policy as a model for potential replication to curb increasing car use in other Chinese cities (Day, 2016). From the theoretical perspective, it is also interesting to find out whether Hong Kong’s built environment characteristics account for a larger variation in commuting travel mode choice, as Hong Kong’s current urban built environment has reached all the 5*Ds* standards that urban planners and researchers have long been looking for. 

This cross-sectional study examines the association of built environment characteristics and commuting mode choice of 203,900 households living in 4768 street blocks in Hong Kong, after adjusting for socioeconomic variables. 

## 2. Methods

### 2.1. The Study Area: Hong Kong

Hong Kong is located in the developed southeastern coastal region of China. It is one of the densest metropolises in the world, with a population of 7.29 million and a gross density of 6603 people per km^2^. Hong Kong encompasses a land area of 1104 km^2^ comprising three major parts: Kowloon, Hong Kong Island, and the New Territories. It is inherently a transit-oriented city characterized by mixed land use and high urban density in most areas because of the land limitation. Hong Kong becomes a microcosm that could be replicated in other Chinese cities with similarly ultra-dense populations and limited land resources [4,31,38].

The citywide public transit system primarily consists of three major modes: the mass transit railway (MTR), trams, and buses. As a successful transit-oriented city, the system provides Hong Kong residents with a high level of mobility (Cervero and Murakami, 2009). The convenient and efficient public transport system, together with the high cost of owning and parking private cars, render the level of car ownership relatively lower than that in other developed cities (0.07 cars per person as of 2016) [39].

### 2.2. Spatial Scales and Unit of Analysis

**Figure 1:**
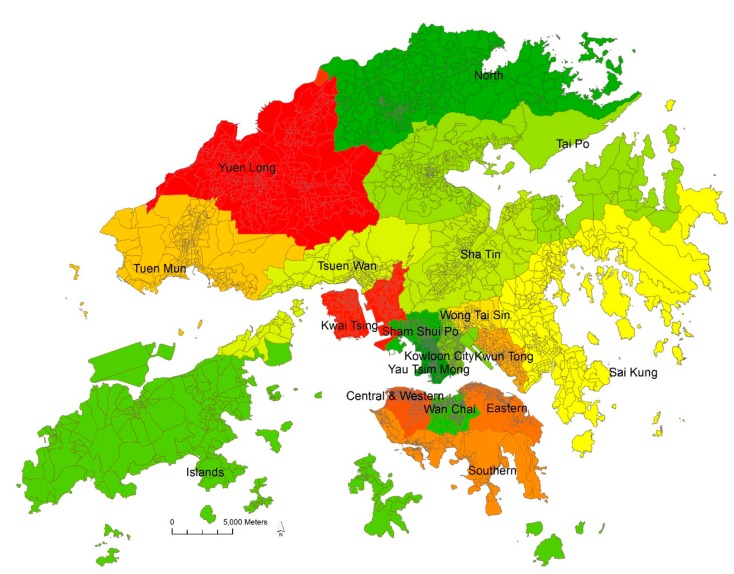
The 18 districts (shown in different colors with district names) and 4768 street blocks (shown as grey lines) in the Hong Kong territory.

The Planning Department of Hong Kong has devised a hierarchical system of planning units at multiple spatial scales—district, town planning unit (TPU), and street block (SB)—covering the whole territory (Figure 1). This structure has also been adopted for disseminating the statistics of population censuses in Hong Kong. There were 18 districts, 289 TPUs, and 4768 SBs in 2011. Each district comprises multiple TPUs, and each TPU comprises multiple SBs. In fact, most street blocks are very small and are made up of one or more housing estates with a homogeneous built environment and socioeconomic characteristics. The 2011 census data show that 90% of street blocks had an area smaller than 0.46 km^2^, and the remaining 10% were rural areas in the New Territories. In this study, we present a street-block-level analysis of the census data for the whole territory of Hong Kong. Street blocks are fine-grained spatial units with adequate detective power to capture the association between commuting mode choice and built environment attributes.

### 2.3. Outcome: Commuting Mode Choice

In 2011 Hong Kong population census, a total of 203,900 households, about one-tenth of all households in Hong Kong, were sampled in a detailed enquiry on demographic and socio-economic characteristics including commuting mode [40]. A multi-modal data collection approach was used: online questionnaire, postal questionnaire, and face-to-face interview. The response rate was 86.3%. The census used stratified sampling method to maintain a sampling fraction of one-on-ten in all smaller planning units, such as SBs and TPUs.

Yet, the mode choice data were available only at Street Block (SB) level, but not at an individual level, from the census. The Census Department aggregated mode choices of all residents living in one SB by showing percentage of three major modes: walking, public transit use (by metro, public, or light buses), and automobile use (by private cars or company cars) [40]. The mode of cycling was excluded from this analysis, because the share of cycling was below 2%, and it was not separately reported in the 2011 census [40]. The census data were collected from the online map service provided by Centamap Company Limited [41].

### 2.4. Built Environment Measures

The built environment of each street block was assessed using the five *Ds* framework. Table 1 shows the definitions of built environment variables examined in this study. All built environmental variables were obtained from 2011 geography information system (GIS) data from Planning Department in Hong Kong.

1. Density. Job density and residential density were assessed as number of jobs and residents per unit area with a street block. This is a typical method of measuring gross density.
2. Diversity. For detailed land use data were not available from the Hong Kong government; number of residents and jobs of different industries (retail, accommodation, and all other jobs) were used to calculate entropy score as a proxy of land-use mix [42]. Land-use mix = (−1) × [(b1/a) ln(b1/a) + (b2/a) ln(b2/a) + (b3/a) ln(b3/a) + (b4/a) ln(b4/a)]/ln (n4), in which b1 = number of residents, b2 = number of retail jobs, b3 = number of accommodation jobs, b4 = number of other jobs, a = total number of residents and jobs, and n4 = 0 through 4 depending on the number of different land uses present.
3. Job−housing balance was also used, which was calculated as employment to population ratio.
4. Design. Street intersection density was defined as the number of intersections (three or more streets) within an 800 m buffer from the centroid of a street block. The 800-m radius buffer was in line with previous studies [43,44,45].
5. Destination accessibility. The distance to CBD was defined as the distance from the centroid of a street block to CBD of Hong Kong (the central area in the Hong Kong Island). The retail density was also assessed, which was defined as the number of supermarkets and convenience stores within an 800 m buffer from the centroid of a street block.
6. Distance to transit stop. The walking distance to closest mass transit railway (MTR) station and number of bus stops within an 800 m buffer from the centroid of a street block were assessed.

### 2.5. Covariates

Besides those built environment factors, residents’ socioeconomic (SES) characteristics have proven to be associated with commuting mode choice in empirical studies. These included median household income, education level (expressed as a percentage of the population aged 15 and over with a college degree), nationality (percentage of Chinese), median age, average household size, proportion of residents working in the same district as their residence, and occupation (proportion of managers and professionals). Those data were also obtained from 2011 census.

### 2.6. Data Analysis

This study used generalized linear mixed models (GLMMs) to study the association of commuting mode choice and built environment characteristics, using SPSS 23 (IBM Corp., Armonk, NY, USA). Given the hierarchical data structure, multi-level modeling with street blocks clustered within districts was used. The multilevel models can examine the association of commuting mode choice and built environment characteristics while adjusting for SES variables; the models can also measure the within-street block and between-district variability in commuting mode choice. The dependent variables are the percentage of three major modes, the value of which bounds between 0 and 1 only. We specified binomial family with a logit link to fit the percentage variables in the GLMMs [46,47]. The models were weighted by number of residents in each street block. To ensure parsimonious fit, initial model building exercise comprised testing for multi-collinearity between the predictor variables through assessment of correlation coefficients and variance inflationary factors (VIF).

Regression coefficients, standardized errors, and p-values are presented as measures of association. We also present the modeling results using elasticity, which allowed us to compare our results with the findings from relatively low-density cities in Western countries [20]. 

## 3. Results

### 3.1. Descriptive Statistics

The descriptive statistics for commuting travel mode choice, the socioeconomic variables, and the built environment variables in a street block were shown in Table 2. On average, at the street block level, 10.5% of residents walked, 66.0% took public transport, and 23.5% used automobiles to commute. 

**Figure 2:**
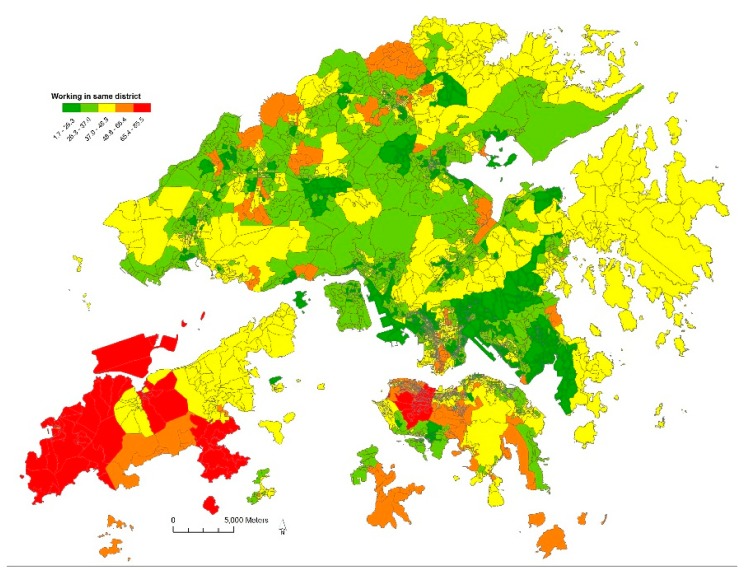
Percentage of residents working in the same district as their residence.

Figure 2 shows the percentage of residents working in the same district as their residence. A recently built new town Tong Chung and the Central-West area of Hong Kong Island had the highest percentage of intra-district travel. Residents living elsewhere needed to commute across districts. The eastern part of Kowloon had the highest percentage of cross-district commuting travel.

**Figure 3:**
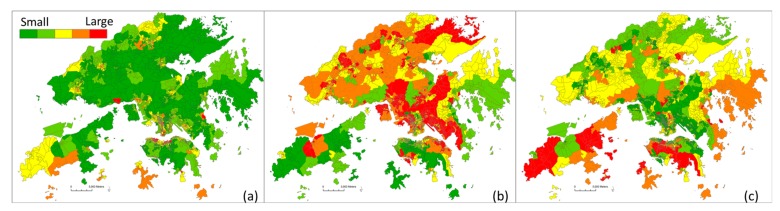
Commuting mode choice for each street block illustrated as a percentage in three categories: walking (**a**), public transit (**b**), and automobile (**c**).

Commuting mode choice in terms of walking, taking public transit, and using automobiles is shown in Figure 3. A few places in Central-West and Kowloon have a slightly higher proportion of people walking to workplaces. Most street blocks in Hong Kong had a relatively low rate of walking. In most places in Kowloon and the New Territories, a significant percentage of residents used public transport to commute. The areas with the lower rates of public transport use were Central-West and Tung Chung. 

### 3.2. Multi-Level Modeling of Travel Choice

The results of two-level mixed effects regression models were presented in Table 3. There is substantial clustering in the model. Interclass correlation coefficient (ICC1) for the walking, public transport, and car travel modes were 28.2%, 38.1%, and 32.5%, respectively, indicating the respective proportion of total outcome variation that is attributed to differences between districts. All variance inflationary factor (VIF) values of multicollinearity test are within 1 and 10, with a maximum value of 8.23 and mean value of 3.50, indicating acceptable levels of collinearity [48]. 

The effect’s magnitudes of the built environment and SES variables on commuting mode choice were presented with elasticity estimates in Table 4. 

*For the mode choice of walking*, socioeconomic characteristics such as household income (elasticity = −0.25), occupation (−0.26), household size (−0.66), and nationality (−0.89) were negatively related to the propensity to walk, while the percentage of residents working in the same district as their residence (0.97) was positively associated with the propensity to walk. A few built environment factors were significantly associated with walking mode, after adjusting for socioeconomic characteristics. Street intersection density (0.33), retail density (0.41), and distance to the urban center (−0.27) had greater elasticity estimate values in absolute terms than the other built environment factors, e.g., land-use mix (0.07) and residential density (0.03). 

*For the mode choice of public transport*, socioeconomic characteristics including household income (elasticity = −0.12), nationality (0.28), and percentage of residents working in the same district as their residence (−0.29) were significantly related to the propensity to use public transport, while median age (−0.05) had the smallest absolute elasticity estimate value. Only distance to the urban center (0.23) had a relatively large elasticity estimate after controlling for socioeconomic characteristics. Other built environment variables had absolute elasticity estimate values of less than 0.1. 

*For the mode choice of driving*, socioeconomic characteristics including household income (0.44), nationality (−0.38), household size (0.23), and working in the same district as their residence (0.37) had larger elasticity estimates in absolute value. Distance to the urban center (−0.50), retail density (−0.20), and distance to metro station (0.12) had absolute elasticity estimate values greater than 0.1, after controlling for socioeconomic characteristics. 

## 4. Discussion

The present study examined the association between commuting mode choice and built environment characteristics in Hong Kong, which is characterized by transit-oriented and high-dense urban from. We found that commuting travel choice was reasonably sustainable in Hong Kong; on average, residents tend to use public transport (66%) for commuting trips more often than driving (24%) or walking (10%). Built environment variables significantly explained the commuting mode choice after adjusting for SES variables, although the order of the variables and magnitude of the effects were different from those reported in previous studies in Western countries.

Specifically, we found that walking to commute was strongly related to design (measured by street intersection density) and destination accessibility (measured by proximity to retail density and urban center). The combined elasticity of walking choice for all built environment factors is large enough to deserve close attention by policymakers and urban planners. The other *Ds*, including the density, diversity, and distance to transit, had insignificant or very modest effects on walking. The elasticity of walking for job−housing balance or land-use mix was very small in absolute terms. Our findings are different from those of the meta-analysis focusing on Western urban contexts, in which walking to commute was strongly related to land-use mix (Ewing, Cervero 2010). The difference may be due to the fact that Hong Kong and many other Chinese cities have already achieved dense development due to a huge urban population. In the same vein, other studies conducted in Chinese cities reported mixed results about of the association of built environment and travel behaviors [33,34,35,36,37]. For instance, residents living in neighborhoods with high urban density walk less than those living in medium urban design in Hong Kong [35]. The number of daily walking trips of old adults was negatively associated with population density and land-use mix in Zhongshan, another dense city in China [33]. Therefore, the policy of densification to promote active living requires further research, especially for identifying the “threshold point” in high-density settings beyond which the benefits of densification become non-significant.

Public transport choice was positively associated with distance to the urban center, which might have been a proxy for proximity of residence to workplace. This result differs from previous findings [20] showing an insignificant effect of distance to the urban center on transit use. Distance to transit stops, measured by bus stop density and distance to the closest metro station, showed small elasticity for taking public transport. This result is different from the findings of Ewing and Cervero’s meta-analysis, which reported greater elasticity for distance to nearest transit stop (0.29) and intersection density (0.23). Both contrasts may be explained by the relatively well-developed urban public transport system in Hong Kong, which runs much more efficiently than most of its counterparts in North America [49]. As shown in Table 2, there are on average 50 bus stops in the 800-m network buffer from the center of a street block in Hong Kong; approximately 58% of street blocks and 70% of residents are within 1 km of an MTR station, and 75% of street blocks and 88% of residents are within 2 km of an MTR station. A lack of the lower end of the spectrum of public transport accessibility may explain the small elasticity of distance to transit stops in our study. 

We found that automobile choice was negatively related to distance to the urban center. The direction of this association is opposite to the findings of Ewing and Cervero, who found that distance to the urban center was positively associated with the propensity to drive. In Hong Kong, residents who are closer to the urban center are more prone to drive to work. Affluent families who have the resources to use a car are clustered in the urban center of Hong Kong, where high-income jobs in banks or law firms and better schools and amenities are concentrated. Soaring housing prices near the urban center have forced the poor to move out. A post-hoc test was conducted by adding a cross-product term between household income and distance to urban center in the regression model; the cross-product term was significant in predicting automobile choice. It indicates significant interaction effect between household income and one built environmental variable. The result may imply that land use policy may affect poor families more strongly than rich families in Hong Kong.

### Limitations and Strengths

This study has several strengths. We provide thorough research for the association between commuting travel patterns and built environment features in Hong Kong, putting this study among the very few studies to focus on high-density cities. The other strengths of the study include its representativeness, its highly detailed measurement of the built environment, and the application of multilevel statistical techniques. The use of census data means that the study achieved extensive population-level and spatial coverage over the entire territory of Hong Kong. It also used detailed spatial modeling to measure the five *Ds* of the built environment. The multilevel modeling strategy ensured robustness in the estimates by considering the hierarchical data structure and the between- and within-neighborhood variability in mode choice.

One of the weaknesses of this study stemmed from its ecological design. Street blocks were the fundamental unit of analysis, and hence we could not adjust for individual-level covariates and socioeconomic characteristics. Nevertheless, street blocks represent the smallest, socially homogeneous, census-defined units, as they are made up of a few housing estates with similar socioeconomic characteristics. Furthermore, this cross-sectional study could not measure the effects of neighborhood self-selection on residents’ preference for a specific commute mode. Prospective studies based on individual-level travel data are needed to further consolidate our findings. We did not unify spatial scales to measure the built environment, but instead used various scales based on data availability. For example, we measured bus stop density using the 800-m walking distance, while job−housing balance was obtained using TPU data. This mismatching of scales created some uncertainty in the built environment assessment. In addition, this study did not measure built environment around workplace or the commuting route, which may also affect commuting mode choice. Yet, the data of workplace location were not collected in the census survey. 

## 5. Conclusions

The built environment characteristics were associated with commuting mode choice in Hong Kong, a dense Asian metropolis known for its sustainable travel behavior. The findings from this study and other studies conducted in China are inconsistent with those from studies conducted in Western countries. This inconsistency suggests that those associations are moderated by local built environment and social contexts. In a high-density urban setting, density and diversity may be ineffective at curbing automobile use in the already extremely compact development. In addition, we suggest that built environment interventions such as pedestrian infrastructure or job−housing balance policies should be implemented together with current fiscal or regulatory policies to stimulate non-motorized travel to alleviate issues, some of which are air pollution, traffic congestion, and residents’ physical inactivity in Hong Kong and other similar high-density cities.

## Figures and Tables

**Table 1:** Built environment measures.

Five Ds Framework	Built Environment Measures	Definition
Density	Job density ^a^	Number of jobs per km^2^ in a TPU
	Residential density	Number of residents per km^2^ in a street block
Diversity	Land-use mix ^a,b^	Entropy score of the number of residents and jobs in different industries (retail, accommodation, and all other jobs) in a TPU
	Job−housing balance ^a^	Ratio of job numbers to the resident numbers in a TPU
Design	Street intersection density	Number of intersections (three-way and above) within an 800-m radius buffer from the centroid of a street block
Destination accessibility	Retail density	Number of supermarkets and convenience stores within an 800-m radius buffer from the centroid of a street block
	Distance to the urban center	Walking distance from the centroid of a street block to urban center (Central in Hong Kong Island)
Distance to transit stop	Distance to MTR station	Walking distance to closest MTR station
	Bus stop density	Number of bus stops within an 800-m radius buffer from the centroid of a street block

^a^ The job data were only available at the TPU level; hence, job density, land-use mix, and job−housing balance were assessed at the TPU level; ^b^ The detailed land use data were not available from the Hong Kong government; hence, the numbers of residents and jobs in different industries (retail, accommodation, and all other jobs) were used as proxy to calculate the land-use mix entropy score [42].

**Table 2:** Descriptive statistics of commuting mode choice, socioeconomic, and built environment characteristics in a street block, using 2011 Hong Kong Census data.

Variables (Unit)	Mean	Standard Deviation	Minimum	Maximum
Commuting mode choice				
Walking (%)	10.4	9.6	0.0	49.7
Public transport (%)	66.0	18.7	3.8	100.0
Car (%)	23.5	19.8	0.0	92.3
SES characteristics				
Household income (HK$1000/month)	33.38	31.58	5.06	256.25
Education (% of college)	30.5	15.6	0.3	87.5
Nationality (% of Chinese)	92.4	12.2	9.8	100.0
Median age	41.95	4.74	25.50	76.70
Occupation (% of mangers or professionals)	39.3	17.11	0.00	87.50
Household size (persons)	2.91	0.53	1.50	5.20
Work in the same district (%)	37.0	13.4	1.7	85.5
Built environment				
Residential density (1000 people/km^2^)	48.56	66.30	0.02	450.50
Job density (1000 jobs/km^2^)	24.03	45.62	0.00	289.60
Job−housing balance (# of jobs /# of populations)	1.53	9.29	0.00	119.06
Land-use mix	0.60	0.31	0.00	1.00
Intersection density (# of intersections in buffer)	120.59	85.04	0	348
Distance to the urban center (km)	11.54	8.46	0.06	40.74
Retail density (# of retails in buffer)	34.17	33.42	0	146
Distance to metro station (km)	1.71	2.37	0.01	23.58
Bus stop density (# of bus stops in buffer)	50.82	41.67	0	163

**Table 3:** Multi-level modeling (generalized linear mixed models) of commuting mode choice.

Variables	Walking	*p* Value	Public Transport	*p* Value	Automobile	*p* Value
	Beta Coefficents (SE)		Beta Coefficents (SE)		Beta Coefficents (SE)	
**SES characteristics**						
Household income	−0.08 (0.01)	<0.01	−0.23 (0.01)	<0.01	0.31 (0.01)	<0.01
Education	0.02 (0.02)	0.13	−0.02 (0.03)	0.45	−0.01 (0.03)	0.81
Nationality	−0.10 (0.01)	<0.01	0.20 (0.02)	<0.01	−0.10 (0.02)	<0.01
Median age	0.02 (0.02)	0.27	−0.08 (0.03)	0.02	0.06 (0.04)	0.10
Occupation	−0.07 (0.01)	<0.01	0.02 (0.02)	0.38	0.05 (0.02)	0.01
Household size	−2.37 (0.23)	<0.01	0.58 (0.42)	0.16	1.83 (0.42)	<0.01
Work in same district	0.27 (0.01)	<0.01	−0.51 (0.02)	<0.01	0.23 (0.02)	<0.01
**Built environment**						
Residential density	0.01 (<0.01)	<0.01	0.01 (<0.01)	<0.01	−0.01 (<0.01)	<0.01
Job density	0.05 (<0.01)	<0.01	−0.02 (<0.01)	<0.01	−0.04 (0.01)	<0.01
Land-use mix	1.16 (0.35)	<0.01	−1.50 (0.62)	0.02	0.33 (0.63)	0.59
Job−housing balance	−0.09 (0.01)	<0.01	0.04 (0.02)	0.06	0.05 (0.02)	0.01
Intersection density	0.03 (<0.01)	<0.01	−0.04 (0.01)	<0.01	0.01 (0.01)	0.03
Dis. to the urban center	−0.24 (<0.01)	<0.01	1.32 (0.07)	<0.01	−1.02 (0.07)	<0.01
Retail density	0.12 (0.01)	<0.01	0.01 (0.01)	0.37	−0.14 (0.01)	<0.01
Distance to MTR	<0.01 (<0.01)	0.95	−1.71 (0.10)	<0.01	1.68 (0.10)	<0.01
Bus stop density	−0.05 (0.01)	<0.01	0.05 (0.01)	<0.01	−0.01 (0.01)	0.63
Household income * Dis. to the urban center	−0.13(0.03)	<0.01	-0.25 (0.02)	<0.01	−0.08 (0.02)	<0.01

**Table 4:** Elasticity estmates for commuting mode choice ^a^.

Variables	Walking	Public Transport	Automobile
**SES characteristics**			
Household income	−0.25	−0.12	0.44
Education			
Nationality	−0.89	0.28	−0.38
Median age		−0.05	
Occupation	−0.26		0.09
Household size	−0.66		0.23
Work in the same district	0.97	−0.29	0.37
**Built environment**			
Job density	0.12	−0.01	−0.04
Residential density	0.03	0.01	−0.03
Land-use mix	0.07	−0.01	
Job−housing balance	−0.01		0.00
Intersection density	0.33	−0.08	0.06
Distance to the urban center	−0.27	0.23	−0.50
Retail density	0.41		−0.20
Distance to metro station		−0.04	0.12
Bus stop density	−0.25	0.04	

^a^ Variables with insignicant elasticity estimates were removed (using *p* < 0.05).
